# The Importance of Rotational Crops for Biodiversity Conservation in Mediterranean Areas

**DOI:** 10.1371/journal.pone.0149323

**Published:** 2016-02-26

**Authors:** Gianpasquale Chiatante, Alberto Meriggi

**Affiliations:** Department of Earth and Environmental Sciences, University of Pavia, Via Ferrata 1, 27100, Pavia, Italy; University of Milan, ITALY

## Abstract

Nowadays we are seeing the largest biodiversity loss since the extinction of the dinosaurs. To conserve biodiversity it is essential to plan protected areas using a prioritization approach, which takes into account the current biodiversity value of the sites. Considering that in the Mediterranean Basin the agro-ecosystems are one of the most important parts of the landscape, the conservation of crops is essential to biodiversity conservation. In the framework of agro-ecosystem conservation, farmland birds play an important role because of their representativeness, and because of their steady decline in the last Century in Western Europe. The main aim of this research was to define if crop dominated landscapes could be useful for biodiversity conservation in a Mediterranean area in which the landscape was modified by humans in the last thousand years and was affected by the important biogeographical phenomenon of *peninsula effect*. To assess this, we identify the hotspots and the coldspots of bird diversity in southern Italy both during the winter and in the breeding season. In particular we used a scoring method, defining a biodiversity value for each cell of a 1-km grid superimposed on the study area, using data collected by fieldwork following a stratified random sampling design. This value was analysed by a multiple linear regression analysis and was predicted in the whole study area. Then we defined the hotspots and the coldspots of the study area as 15% of the cells with higher and lower value of biodiversity, respectively. Finally, we used GAP analysis to compare hotspot distribution with the current network of protected areas. This study showed that the winter hotspots of bird diversity were associated with marshes and water bodies, shrublands, and irrigated crops, whilst the breeding hotspots were associated with more natural areas (e.g. transitional wood/shrubs), such as open areas (natural grasslands, pastures and not irrigated crops). Moreover, the results underlined the negative effects of permanent crops, such as vineyards, olive groves, and orchards, in particular during the winter season. This research highlights the importance of farmland areas mainly for wintering species and the importance of open areas for breeding species in the Mediterranean Basin. This may be true even when the species’ spatial distribution could be affected by biogeography. An important result showed that the hotspots for breeding species cannot be used as a surrogate for the wintering species, which were often not considered in the planning of protected areas.

## Introduction

Biodiversity preservation and restoration are the most important goals of conservation biology, and nature reserves play a vital role in achieving this goal [1]. However, the effectiveness of protected areas in representing biodiversity has been frequently questioned [2–4], and it is accepted that existing conservation areas usually provide inadequate coverage to biodiversity [5–10]. Thus, selection of critical areas for biodiversity conservation needs to prioritize areas on the basis of their biodiversity value, selecting those that have the highest priority, and needs to set precise prescriptions [11–15].

In Italy, conservation measures and the selection of protected areas have always been localized in areas characterized by low levels of human presence and intervention [16]. In particular, Italian protected areas tend to over-represent mountainous areas and other regions with low economic values, while the coverage offered by protected areas in the Mediterranean part is limited [16]. As Maiorano et al. [16] stated, there is a clear indication that the existing protected areas cannot be considered to be fully representative of Italian vertebrate biodiversity. Besides, Italy and the Mediterranean basin have seen thousands of years of intense human presence, with a complex integration of traditional human activities and natural ecosystems leading to high environmental diversity and also to high fragmentation. As a result, a complex and ecologically rich cultural landscape has formed [17].

Thus, in the Mediterranean region more than anywhere else, the protected areas must be planned and managed in conjunction with the traditional agricultural and husbandry activities, and the only viable option for conservation is that of considering human presence and human activities as an integral part of the system [16,18,19].

The main goal of this research was to define if a crop dominated landscape represents an important context for bird diversity conservation, in order to prioritize areas for conservation purposes. To this end, we identify the hotspots and the coldspots of bird diversity in central Apulia (southern Italy), both during the winter and the breeding season. The landscape of this very poorly known region is dominated by agro-ecosystems, and it is inside one of the “hottest hotspots” of the world, the Mediterranean Basin [12,20,21].

Farmland bird species represent a large proportion of European avifauna, and the populations of several of those species have suffered a dramatic decline in the last decades, especially in Western Europe [22,23]. The causes of this decline have been identified mostly as changes of agricultural practices, such as heavy mechanization, input increase, temporal shift of cereal sowing from spring to autumn, and loss of landscape heterogeneity determined by the destruction of hedgerows, shrub and tree patches and other natural areas, which follows intensification [22–26]. These changes led both to reduction of refuge and reproduction areas and to decrease of invertebrate prey, the latter largely prompted by the increase in biocide use [23,27–29]. A further cause of farmland species’ decline is represented by land abandonment [22,30,31], which is now threatening important farmland bird populations in mountain areas [32].

In addition, this study could be interesting because of biogeographical aspects. Indeed, within the study area the *peninsular effect* is evident which leads to a decline in species richness as a function of distance from the mainland toward the distal tip of a peninsula [33–35]. Because of its geographical position the Apulia region could be defined as *a peninsula within a peninsula*, so the spatial pattern of bird species could be affected by this effect. Therefore, testing farmland bird response to agro-ecosystem features in a biogeographically interesting context in the Mediterranean Basin hotspot may potentially contribute to increase practical knowledge for a prioritised conservation issue. Other research aims were (i) to establish if hotspots of breeding birds can be used as surrogates of wintering bird hotspots and *vice versa*, and (ii) to compare the hotspots and the coldspots with the current protected area network of the region. We expected that a landscape dominated by agro-ecosystems could be an important and interesting framework for biodiversity maintenance, even when the species distribution could be affected not only by human modifications but also by biogeography. We also expected different hotspots for wintering and breeding birds, and a good cover of protected areas, though only for breeding bird hotspots.

## Materials and Methods

### Study area

**Fig 1:**
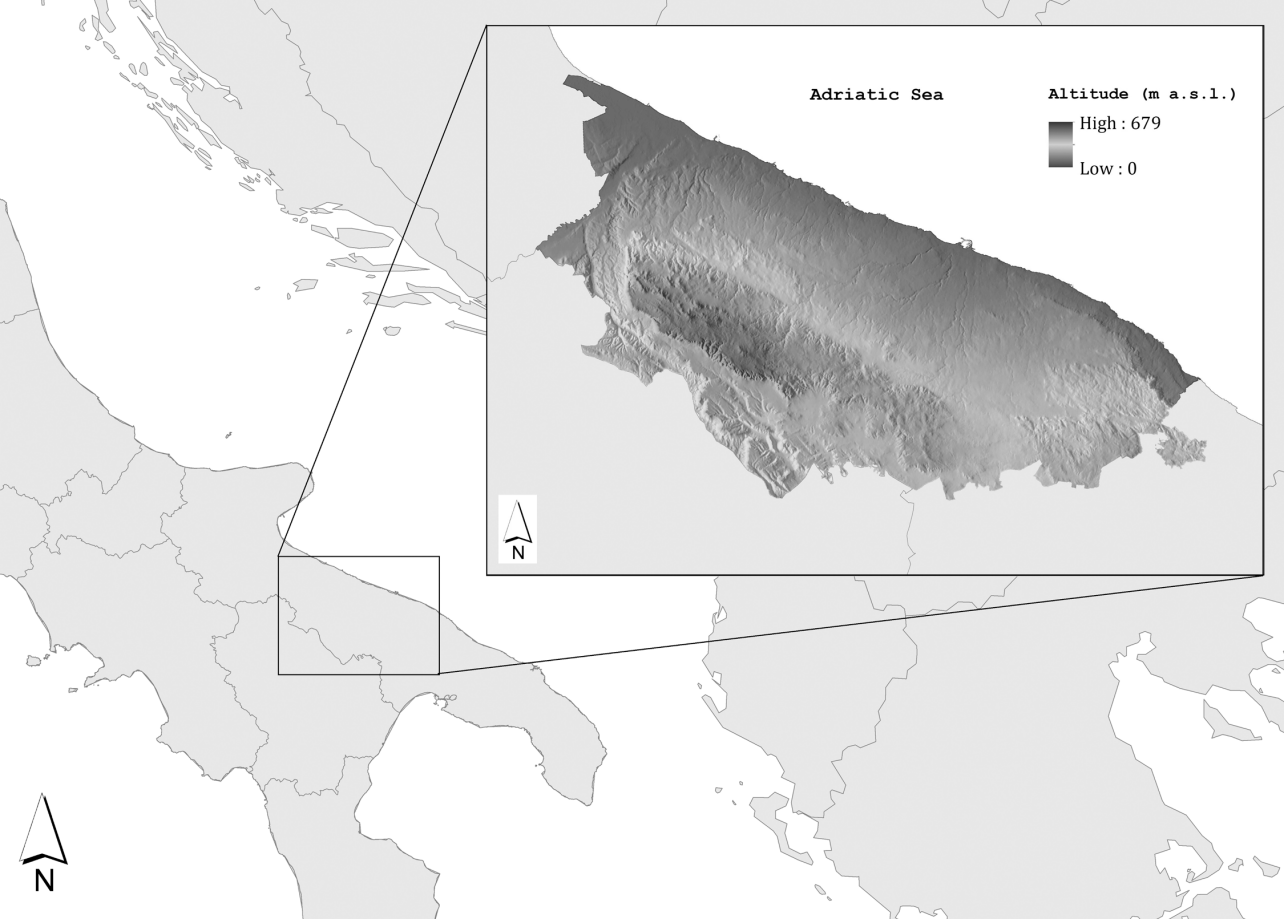
The study area in Central Apulia (southern Italy).

The study area occupies the central part of the Apulia region in southern Italy (N 41°0’ E 16°34’), more precisely the Bari and Barletta-Andria-Trani provinces, over an area of 5406 km^2^ (Fig 1). The altitude ranges from sea level to 679 m a.s.l. (Mt. Caccia), with 35% of the altitude comprised between sea level and 200 m a.s.l. and 40% between 201 and 400 m a.s.l. The climate is typically Mediterranean: along the coastal side and in the lowlands the summers are warm, windy, and dry, whilst the winters are mild and rainy. The minimum temperature varies between 2–5°C in January-February and 16–19°C in July-August. The maximum temperature varies between 10–13°C in January-February and 28–30°C in July-August. Precipitation, concentrated during the late autumn and winter, is scarce and in the form of rain. The average values of rainfall vary between 27–28 mm in July and 67 mm in October. The landscape is characterized mainly by not irrigated cereal crops (32.4%) and olive groves (27.6%). Vineyards represent 8.7% of the surface, followed by urban areas (8.5%), grasslands, pastures and fallows (7.5%), orchards (5.7%), and forests (4.5%). The study area comprised 51 municipalities with a total resident population of 1,638,743 (ISTAT data, 2013) and a density equal to 303 per km^2^. In the study area there are 18 protected areas which extend over 978 km^2^ (18.1% of the study area). Moreover, there are 9 Sites of Community Importance (1595 km^2^, 29.5% of the study area) and 2 Special Protection Areas (1296 km^2^, 24.0% of the study area).

### Sampling design

To collect data for identifying the bird diversity hotspots we used a stratified random sampling design, with proportional allocation of samples to guarantee the same sampling effort in each stratum [36,37]. Thus, we partitioned the study area into 5655 squares of size 1 km^2^. Then we identified homogeneous areas, referred to as landscape units (LU) by a clustering of similar squares by the help of k-means cluster analysis [37–39]. To this purpose we measured in each cell the percentages of land use types by a geographic information system (GIS) platform (ArcGIS 10.2.1, ESRI, Redlands, CA) using a regional land use map at fourth level of CORINE land cover 1:5000 (2011 update, SIT–Regione Puglia; S1 Table). Then we tested the goodness of the LU classification by the non parametric Kruskal-Wallis test and Discriminant Function Analysis on the same environmental variables used for clustering [39].

### Field data collection

To collect data for the *wintering hotspots of bird diversity* we carried out linear transects (*ca*. 500, 1000, and 2000 m long) randomly placed in the study area and in number proportional to LU extension [37,40]. Transects were straight lines randomly placed in the LU both in the starting point and in direction using the DNR Sampling Tool v2.8 extension for ArcView 3.2 (Minnesota Department of Natural Resources). In particular, 264 transects (S2 Table) were walked once during December and January of the wintering seasons from 2011 to 2014 (2011–2012: 110 transects; 2012–2013: 91 transects; 2013–2014: 63 transects). To collect data on *breeding hotspots of bird diversity*, we carried out call counts in 301 hearing points randomly placed in the study area according to the stratified sampling between late April and early June of the breeding season 2012 (170 sampling points) and 2013 (131 sampling points) (see S2 Table) [40–42]. Each point was surveyed once from dawn to 11:00 and the count lasted for 10 minutes [42,43]. A species was considered a breeder if it was observed at least in territorial behavior, such as song. In this way it was possible to discard the migratory species. Surveys were not conducted on windy or rainy days. We used a binocular Vortex Viper 10x40 and a GPS tracker Garmin eTrex Venture to position the observations in the area. For the nomenclature we followed the International Ornithologists’ Union list [44].

The effectiveness of the sampling design was assessed comparing the observed number of species in each cluster with the expected species number obtained by the non parametric Chao Index [43,45]. In particular, if the number of observed species was included in the 95% confidence interval of expected species the sampling design and the sampling effort were considered appropriate.

### Statistical analysis

To identify the bird diversity hotspots we used a method proposed by Rey Benayas and de la Montaña [46]. In particular, for each point or transect (precisely its geometric centroid) carried out in the fieldwork, we calculated four parameters to identify areas of high-value of bird diversity: 1) the *species richness S*_*r*,_, i.e. the number of bird species occurring in each sample. 2) The *rarity index R*_*r*_, defined by the species geographical range measured as the inverse of the number of samples where it was present (1/n_*i*_); for a sample *r*, the rarity index was Σ_*i = 1*_ (1/*n*_*ri*_)/*S*_*r*_, where *S*_*r*_ was the species richness in the sample *r*. 3) The *vulnerability index V*_*r*_, quantified using the categories of the *Red List of Italian Breeding Birds* [47]. A score was assigned to every species related to its degree of vulnerability: 3 for *Endangered* (EN), 2 for *Vulnerable* (VU) and *Near Threatened* (NT) species, 1 for *Least Concern* (LC) species, 0 for *Alien* and *Data Deficient* (DD) species. Moreover, if the species was listed in the Annex I of the *Birds Directive* 2009/147/CE a value of 1 it was added, and considering the SPEC categories (*Species of European Conservation Concern*) suggested by BirdLife International [48], 1.5 was added for SPEC 1 (species of global conservation concern), 1 for SPEC 2 (species whose world populations are concentrated in Europe and which have an unfavorable conservation status), and 0.5 for SPEC 3 (species whose world populations are not concentrated in Europe, but which have an unfavorable conservation status in Europe). For a sample *r*, the vulnerability index was Σ_*i = 1*_ *V*_*ri*_/*S*_*r*_, where *V*_*ri*_ was the vulnerability score of the species *i* present in the sample *r*. 4) The *combined index* of bird diversity *C*_*r*_, which summarized the species richness, the rarity index, and the vulnerability index, calculated for the sample *r* as Σ_*i = 1*_ (1/*n*_*ri*_) *V*_*ri*_.

We used the combined index obtained in each sample to predict its value on the whole study area both during the winter and the breeding season formulating a Multiple Linear Regression Analysis (MLRA) [39]. In particular we investigated the relationships between the combined index and the percentage cover of land use classes (land use map at the 4^th^ level of CORINE 1:5000, SIT–Regione Puglia, Table 1) measured in the 1 km^2^ cells in which the samples where located. When more than one sample falls in the cells, we used the mean values of their combined index.

**Table 1:** Land use classes with significant differences between the Landscape Units obtained by the cluster analysis (Kruskal-Wallis test).

Land use variables (% cover)	*H*	*df*	*P*
Urban areas	1493.7	9	< 0.001
Extractive areas	125.4	9	< 0.001
Irrigated crops	2497.1	9	<0.001
Not irrigated crops	4018.8	9	< 0.001
Horticulture	193.0	9	< 0.001
Vineyards	2688.2	9	< 0.001
Orchards	1464.1	9	< 0.001
Olive groves	3912.9	9	< 0.001
Meadows	166.1	9	< 0.001
Annual crops associated with permanent crops	393.7	9	< 0.001
Complex cultivation patterns	223.4	9	< 0.001
Arable lands with natural vegetation	23.2	9	0.006
Woodland	1560.0	9	< 0.001
Pastures and meadows with scattered trees	339.8	9	< 0.001
Pastures and natural grasslands	1473.2	9	< 0.001
Shrublands and Mediterranean maquis	274.7	9	< 0.001
Transitional wood/shrubs	179.8	9	< 0.001
Beaches, dunes, sands	219.6	9	< 0001
Bare soils and rocks	268.7	9	< 0.001
Sparsely vegetated areas	200.2	9	< 0.001
Marshes, rivers, water bodies	485.8	9	< 0.001
Saltmarshes and salt flats	3297.4	9	< 0.001

Considering the non normality of the dependent variable both during the winter (Kolmogorov-Smirnov test, *P* < 0.001) and the breeding season (Kolmogorov-Smirnov test, *P* < 0.001), we used the logarithmic transformation making it normal (Kolmogorov-Smirnov test, *P*_*wintering*_ = 0.250, *P*_*breeding*_ = 0.133) [39,49].

We simplified the model following a backward stepwise approach using an Information Theoretic Approach [50] selecting the variables by the Akaike Information Criterion (AIC, [51]. The Variance Inflation Factor was measured for the model with a threshold of 3, to test the variables’ collinearity [52–54]. The goodness-of-fit of the predicted values and the observed ones was tested by the Pearson’s correlation test [39]. Moreover we tested the residuals for normality by the Kolmogorov-Smirnov test [39] and for independence by the Durbin-Watson test [55,56].

Finally, we used the predicted values of the combined index in each cell of the 1 km spaced grid to identify the biodiversity hotspots and coldspots. More precisely, we classified as bird diversity hotspots the 15% of the cells (848 cells) with the highest value of the combined index and as coldspots the 15% of the cells (848 cells) with the lowest values of this index [46].

### Hotspots and protected areas

To define the protected surface of the study area, we considered each 1 km^2^ cell as currently “protected” when at least 50% of its surface was covered by a protected area [57]. To establish the protected area network, every type of protection was accounted for, the Natura 2000 Network (Special Protection Areas and Sites of Community Importance) comprised. Then, we measured how the existing protected areas represented the bird diversity (both wintering and breeding hotspots). In particular we compared through the Mann-Whitney *U* test [39] the combined index between protected cells and hotspot cells and correlated the percentage of protected areas in the 5655 cells and the combined index by Pearson’s correlation test, both during the wintering and the breeding season. Then, to establish the concordance between the hotspots and the protected areas, the Cohen Kappa statistic for agreement was calculated [58,59]. Kappa statistic ranges between 0, when there is no agreement between the cases, to 1, when there is a complete agreement between the cases [60]. Finally, to calculate the overlap between protected areas and hotspots, GAP analysis was used [61,62].

### Ethics statement

This research was conducted with ethical approval from the University of Pavia (Department of Earth and Environmental Sciences). Bird surveys were conducted with permission from local landowners where necessary. Data collection did not involve sampling procedure and experimental manipulation of birds and the field work was conducted under the Law of the Republic of Italy on the Protection of Wildlife (February 25, 1992).

## Results

### Habitat classification

Considering the land use types the 5655 cells were grouped in ten groups of homogeneous Landscape Units (LU). The Kruskal-Wallis test showed that the most representative land use types in the study area differed significantly between the Landscape Units (Table 1). The canonical correlations of the Discriminant Function Analysis were highly significant (*P* < 0.001) and 94.8% of the cells were correctly reclassified in the resulting clusters. For the description of Landscape Units obtained see S3 Table.

### Bird diversity during the winter and the breeding season

The non parametric Chao Index showed that both during the winter and the breeding season the observed number of species in each LU was comparable to that expected, so the sampling effort could be considered good (Table 2).

**Table 2:** Observed and expected species number (95% confidence intervals) of each LU obtained by non parametric Chao Index.

LU	Linear transects		Point counts	
	Observed	Expected	Observed	Expected
1	45	55 (40–69)	22	29 (15–44)
2	24	26 (22–30)	10	-
3	53	66 (42–90)	38	46 (31–60)
4	46	58 (41–74)	38	48 (32–63)
5	61	83 (56–110)	54	96 (32–160)
6	43	54 (36–72)	40	49 (34–65)
7	41	51 (34–68)	29	34 (25–44)
8	45	49 (41–57)	34	40 (29–51)
9	38	44 (33–55)	40	73 (33–113)
10	53	59 (49–68)	55	59 (52–66)

During the winter season we recorded 124 species in the study area (S4 Table), out of which 24 species (19.4%) are listed in the Annex I of the *Bird Directive* 2009/147/CE, 31 species (25.0%) are listed in the *Red List of Italian Breeding Birds* as endangered (5 species), vulnerable (16 species) or near threatened (10 species), and 43 species (34.7%) are SPEC (SPEC 1 = 1 species, SPEC 2 = 14 species, SPEC 3 = 28 species). Considering the total number of observations in the wintering season (*N* = 9657), the most common species was *Pica pica* (7.5%, *N* = 721), followed by *Fringilla coelebs* (7.1%, *N* = 686), *Erithacus rubecula* (7.1%, *N* = 683), *Serinus serinus* (3.8%, *N* = 367), *Cyanistes caeruleus* (2.8%, *N* = 268), *Sylvia melanocephala* (2.7%, *N* = 262), *Turdus philomelos* (2.7%, *N* = 261), *Parus major* (2.7%, *N* = 259), and *Passer italiae* (2.7%, *N* = 257). The most rare species (0.01%, *N* = 1) were *Anas acuta*, *Platarea leucorodia*, *Falco columbarius*, *Rallus aquaticus*, *Recurvirostra avosetta*, *Calidris alba*, *Calidris minuta*, *Philomachus pugnax*, *Limosa limosa*, *Tringa glareola*, *Larus audouinii*, *Anthus spinoletta*, *Remiz pendulinus*, and *Lanius excubitor*.

During the breeding season we recorded 108 species in the study area (S4 Table), out of which 27 species (25.0%) are listed in the Annex I of the *Bird Directive* 2009/147/CE, 35 species (32.4%) are listed in the *Red List of Italian Breeding Birds* as endangered (8 species), vulnerable (19 species) or near threatened (8 species), and 46 species (42.6%) are SPEC (SPEC 1 = 1 species, SPEC 2 = 13 species, SPEC 3 = 32 species). Considering the total number of observations in the breeding season (*N* = 8762), the most common species was *P*. *pica* (10.0%, *N* = 878), followed by *P*. *italiae* (6.2%, *N* = 545), *Emberiza calandra* (5.1%, *N* = 449), *Galerida cristata* (5.0%, *N* = 440), *P*. *major* (4.2%, *N* = 370), *S*. *serinus* (4.1%, *N* = 362), *Hirundo rustica* (3.7%, *N* = 320), *Passer montanus* (3.5%, *N* = 306), and *Streptopelia decaocto* (3.2%, *N* = 277). The most rare species (0.01%, *N* = 1) were *Tadorna tadorna*, *Anas platyrhynchos*, *Ixobrychus minutus*, *Fulica atra*, *Glareola pratincola*, *Larus melanocephalus*, *Larus michahellis*, *Gelochelidon nilotica*, *Sterna sandvicensis*, *Motacilla cinerea*, *Riparia riparia*, *Phylloscopus collybita*, and *Emberiza melanocephala*.

### Wintering hotspots of bird diversity

Considering the species observed during the winter season, we calculated the four indexes for each sample. The species richness ranged from 1 to 35 species (mean ± SE: 11.5 ± 0.31), the rarity index ranged from 0.007 to 0.46 (mean ± SE: 0.02 ± 0.002), the vulnerability index ranged from 0.67 to 2.5 (mean ± SE: 1.29 ± 0.01), and the combined index ranged from 0.02 to 8.50 (mean ± SE: 0.37 ± 0.04).

Four land use variables entered the best regression model obtained by AIC selection (Table 3). The most reliable effects were the positive one of marshes, rivers, water bodies, and irrigated crops, and the negative one of olive groves. The positive effects of shrublands were not so reliable because their confidence interval encompassed the 0 value. There was no collinearity between variables (VIF < 3) and the goodness-of-fit was fair (*r* = 0.354, *P* < 0.001). The residuals were normally distributed (Kolmogorov-Smirnov test, *D* = 0.05, *P* = 0.518) and independent (Durbin-Watson test, *DW* = 1.91, *P* = 0.243).

**Table 3:** The best model obtained by MLRA for the wintering hotspots of bird diversity.

Variables	β	SE	LCI 95%	UCI 95%	VIF
Intercept	-1.433	0.059	-	-	-
Irrigated crops	0.010	0.005	0.001	0.019	1.01
Olive groves	-0.008	0.002	-0.011	-0.004	1.02
Shrublands	0.023	0.014	-0.005	0.052	1.01
Marshes, rivers, water bodies	0.064	0.020	0.026	0.103	1.00

β = model estimate, SE = standard error, LCI 95% = 95% lower confidence interval, UCI 95% = 95% upper confidence interval, VIF = Variance Inflation Factor.

**Fig 2:**
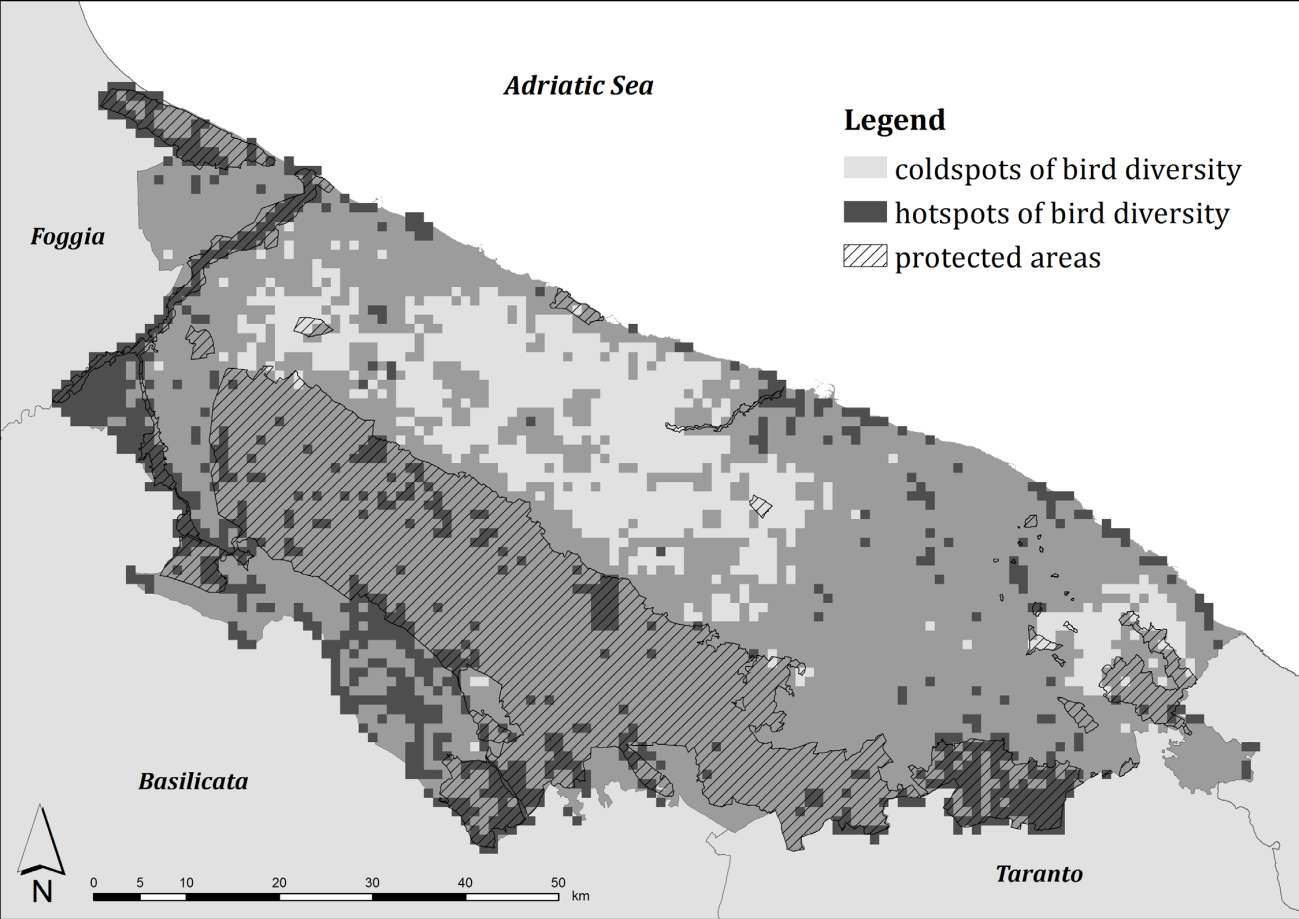
Wintering hotspots of bird diversity in central Apulia and protected area boundaries.

In the study area there were many wintering hotspots of bird diversity, localized mainly in the north (in the Margherita di Savoia salt flats and along the Ofanto and Locone rivers), in the west (along the boundary with the Basilicata region), and in the south (along the boundary with Taranto province). In contrast, two main coldspots were predicted: the biggest in the central and coastal part of the study area, the smallest in south-eastern part (Fig 2).

### Breeding hotspots of bird diversity

Considering the species observed during the breeding season, the species richness ranged from 0 to 17 (mean ± SE: 8.3 ± 0.17), the rarity index ranged from 0 to 0.46 (mean ± SE: 0.03 ± 0.003), the vulnerability index ranged from 0 to 3 (mean ± SE: 1.62 ± 0.02), and the combined index ranged from 0 to 12.35 (mean ± SE: 0.42 ± 0.05).

Five land use variables entered the best regression model obtained by AIC selection (Table 4). The most reliable effects were the positive one of transitional wood/shrubs and the negative one of orchards and olive groves. The negative effects of urban areas and vineyards were not so reliable because their confidence interval encompassed the 0 value. There was no collinearity between variables (VIF < 3) and the goodness-of-fit was fair (*r* = 0.399, *P* < 0.001). The residuals were normally distributed (Kolmogorov-Smirnov test, *D* = 0.07, *P* = 0.147) and independent (Durbin-Watson test, *DW* = 1.91, *P* = 0.198).

**Table 4:** The best model obtained by MLRA for the breeding hotspots of bird diversity.

Variables	β	SE	LCI 95%	UCI 95%	VIF
Intercept	-1.096	0.085	-	-	-
Urban areas	-0.006	0.003	-0.012	0.001	1.04
Transitional wood/shrubs	0.050	0.022	0.006	0.093	1.02
Vineyards	-0.006	0.003	-0.013	0.001	1.02
Olive groves	-0.009	0.002	-0.012	-0.005	1.03
Orchards	-0.015	0.004	-0.023	-0.008	1.02

β = model averaged coefficient, SE = standard error, LCI 95% = 95% lower confidence interval, UCI 95% = 95% upper confidence interval, VIF = Variance Inflation Factor.

**Fig 3:**
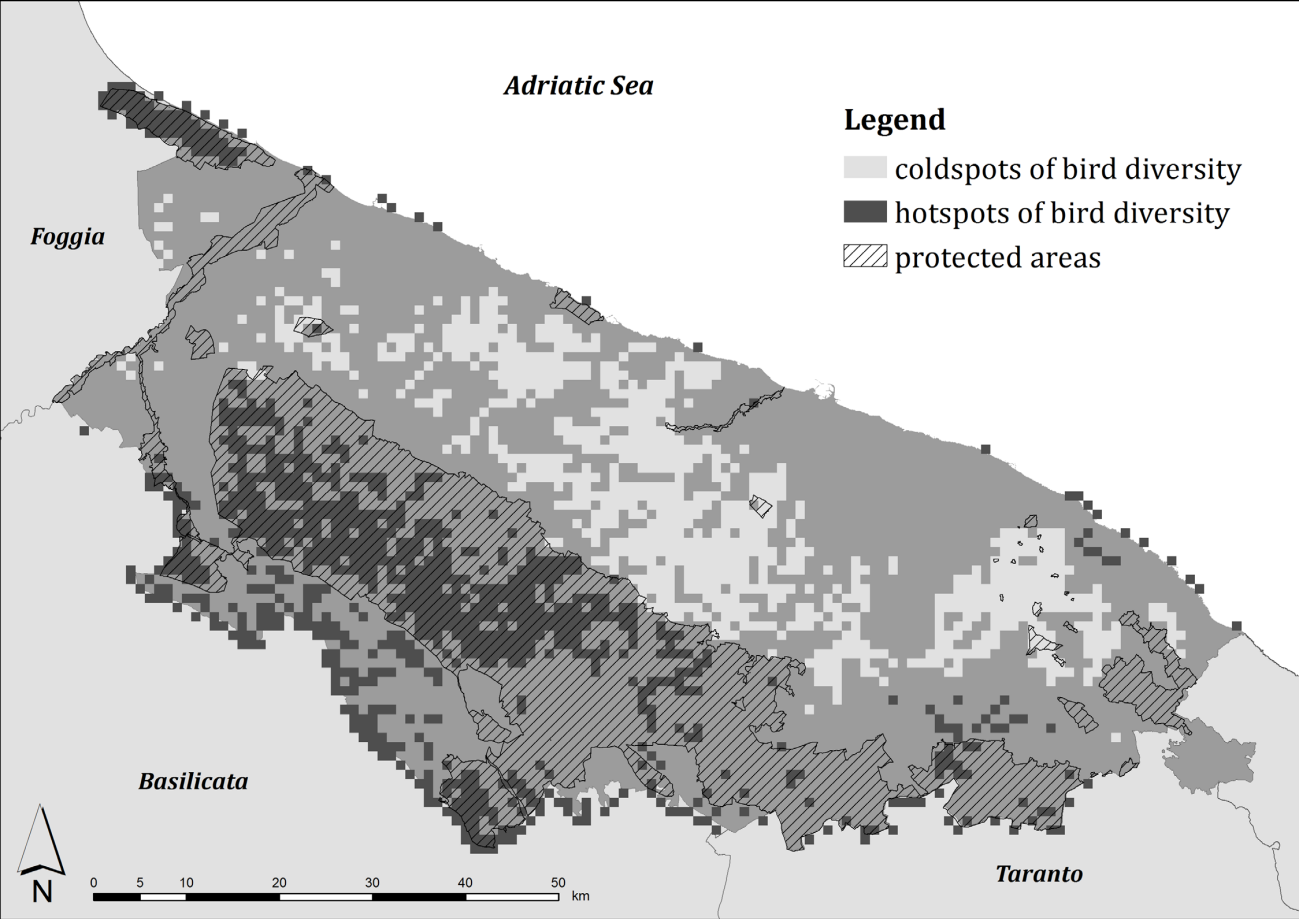
Breeding hotspots of bird diversity in central Apulia and protected area boundaries.

In the study area breeding hotspots of bird diversity resulted in the north (the Margherita di Savoia salt flats), in the center (corresponding to the Alta Murgia Plateau), and in the west (along the boundary with Basilicata region). In addition, coldspots were localized mainly along the Adriatic Sea coast (Fig 3).

### Hotspots and protected areas

The cells falling in protected areas had the combined index higher than the hotspot cells both during the winter (*U* = 1,295,338, *P* < 0.001) and the breeding seasons (*U* = 1,207,084, *P* < 0.001) such that the percentage of protected areas was positively correlated with the combined index both during the winter (*r* = 0.388, n = 5655, *P* < 0.001) and the breeding season (*r* = 0.571, n = 5655, *P* < 0.001). Nevertheless, the Kappa statistic was equal to 0.125 and to 0.314 during the winter and the breeding season respectively, showing a slight and a fair agreement between the cells considered as hotspots and the cells falling in protected areas. The GAP analysis showed that 45.9% of the area considered as wintering hotspots was inside protected areas while 68.7% of the area considered as breeding hotspots was protected. In contrast, only the 2.1% and 1.3% of land considered as coldspots were included in protected areas.

## Discussion

The main goal of this research was to define if crop dominated landscapes could be useful for bird diversity conservation, both in the winter and in the breeding season, in a biogeographically important area of the Mediterranean Basin. In the study area the species richness was greater during the winter than during the breeding season (77 *vs* 72 species respectively). Contrasting results were found in another Mediterranean study area, where the species richness was greater during the breeding season than in the wintering one [57]. The field data collection support the *peninsula effect* because many common species in the mainland of Italy (i.e. *Columba palumbus*, *Cuculus canorus*, *Picus viridis*, *Dendrocopos major*, *Delichon urbicum*, *Troglodytes troglodytes*, *E*. *rubecula*, *Luscinia megarhynchos*, and *Turdus merula*) [63] are very scarce as breeders in the study area. Our results showed that during the winter higher bird diversity was found in irrigated crops and marshes, rivers, and water bodies, while lesser diversity was found in olive groves. On the other hand, during the breeding season the highest values were found in transitional wood/shrubs areas, while the lowest were found in permanent crops, such as olive groves and orchards. Considering that not irrigated crops are the most important land use in the study area (32.4%), these negative effects underlined the importance of this habitat, as also showed by the importance for breeding birds of the Alta Murgia Plateau (in the central part of the study area and comprised in the Alta Murgia National Park), one of the largest steppe areas of Italy. This importance was noted, mainly for larks, by a recent research carried out in this area [64]. Further, the importance of wetlands for wintering birds is usual in the Mediterranean Basin [65–68] and the Margherita di Savoia salt flats are one of the most important wintering areas for waterbirds in Italy [69,70].

The major differences between the two seasons were in the selection of more crop dominated areas during the wintering and more natural areas during the breeding season. This was due to the fact that during the winter, migrant species more related with humans and agricultural land come into the study area, such as *Motacilla alba*, *E*. *rubecula*, *T*. *merula*, *T*. *philomelos*, *Sylvia atricapilla*, *Sturnus vulgaris*, *F*. *coelebs*, *S*. *serinus*, and *Linaria cannabina*. On the other hand, during the breeding season, more migrant steppe species (*i*.*e*. *Falco naumanni*, *Coracias garrulus*, *Melanocorypha calandra*, *Calandrella brachydactyla*, *Anthus campestris*, *Oenanthe hispanica*, *Sylvia conspicillata*, *Lanius minor*, *Lanius senator*, and *E*. *calandra*) come into the study area. Higher species richness during the breeding season was found in open areas in Spain [71] as well as outside of the Western Palearctic [72]. In contrast, in Atlantic France cereal crops did not favor species richness [73]. Moreover, in the present study we found a positive effect of transitional wood/shrubs areas on bird diversity. Similar results were found in Spain and in France, where greater species richness was observed during the breeding season in woodlands and shrublands [71,73–75]. These results were in concordance with a general rule of breeding birds in the Mediterranean Basin, where the highest number of species are found in steppes (25.7%) and in forests (24.3%) [20]. In general, olive groves, in particular the winter fruits, provide important resources for birds [76,77], such as *E*. *rubecula*, *T*. *merula*, *T*. *philomelos*, *S*. *atricapilla*, *S*. *melanocephala*, and *S*. *vulgaris*, very abundant in the study area during the wintering season. The negative effect of olive groves on species diversity during the winter and the large winter coldspot in the north of the study area, where olive groves were present, could be due to the fact that the herbaceous ground cover, which affects positively the species richness providing seeds and insect prey for foraging birds [78], was usually absent, because of the intensive agricultural practices (*i*.*e*. the use of herbicides and the frequent soil harrowing).

Olive groves and orchards hosted more widespread and generalist species, while in the transitional wood/shrubs areas, pastures, natural grasslands, and in the not irrigated crops there were more localized and specialized species. Furthermore, the importance of the Alta Murgia plateau highlighted the positive relationships between open areas and the more vulnerable species according to international and national lists. This relationship agrees with the findings of other studies, which pose the bird species of open areas as the most threatened in Mediterranean Europe [79,80].

This research showed that bird conservation can also be done in crop-dominated landscapes, where the best areas for the wintering and breeding birds are located. This was true in the Mediterranean regions, where traditional agricultural landscapes and extensively managed mosaics are characteristic [81–83]. The importance of agricultural land in the protected areas network was highlighted in other researches [9,18,74,84]. In order to render agricultural landscapes efficient in complementing protected areas, agro-environmental practices that increase landscape heterogeneity and structural complexity should be emphasized [23,75,85–87]. These may include mixed farming and the presence of natural vegetation in field margins, hedgerows, or in-field strips [88]. Improving the management of open and agricultural habitats within protected areas, could be a good way to achieve the goal of farmland bird conservation [24,25,89–91].

Identifying gaps in the representation of species in protected areas, although important, was only one step, among others, toward building more effective networks of protected areas for conservation [11]. Designing boundaries for protected areas would require fine resolution species data (currently unavailable for most *taxa*) or the downscaling of individual species distributions [92]. In this view, the approach applied in this research to use a 1-km grid cell was very advantageous both to identify high value areas and to compare them with the current protected areas. Other studies have used a more coarse resolution, with larger grids, even if the researches were conducted at regional or national levels [46,93–97]. A complete design would also include socioeconomic information to estimate management, acquisition, and opportunity costs associated with the implementation of conservation programs [98–100].

## Conclusion

In this research we showed the importance of crop dominated landscapes in the Mediterranean Basin for bird diversity conservation. In particular we highlighted the importance of open areas, such as natural grasslands, pastures, and not irrigated cereal crops, for conservation of breeding birds. Conversely, other researches provide insights that open habitat and habitat homogenization could be deleterious for bird diversity [26,73], even though the study areas investigated were not sited in Mediterranean regions. Moreover, the hotspots for breeding birds cannot be used as a surrogate for the wintering bird species, as observed in other places of the Mediterranean Basin and of the world [57,101]. Thus, the need to plan the protected areas network to take into account the wintering species is emphasized as well. Many studies have helped to establish priority conservation areas based only on the distribution of breeding bird species [9,18,46,85,102–104]. Nevertheless, worldwide, few studies have dealt with wintering species distribution in order to evaluate the effectiveness of conservation reserves [71,105,106]. Likewise, research indicates that protection of non-breeding habitats may be crucial to avian conservation, because mortality was exacerbated in this period [66,107–109] and many species appear to be primarily constrained by survival on their wintering grounds [110,111]. Petit [112] found that some habitats which were relatively unoccupied during the breeding season become important during the winter. Furthermore, large-scale conservation plans should consider other taxonomic groups, not only avian species, so that the final design of the protected areas network should cover the complete scope of biodiversity. This was true, because of the overlap of hotspots between different *taxa* was generally low, especially when groups have very different ecological requirements; this mismatch has been reported for many *taxa* in different parts of the world [95,113–115]. Therefore a strategy for protected areas designation based solely on a few limited numbers of *taxa* may fail to provide adequate protection for many other organisms [116–120].

## Supporting Information

S1 Table Land use variables used for cluster analysis and as predictors in the multiple linear regression to assessing bird diversity hotspots in Southern Italy. (DOCX)

S2 Table Landscape Units (LU), length of transects and number of point counts carried out in the research. Density of sampling transects (1 km/km^2^) and sampling points (1 point/km^2^) are showed as well.
(DOCX)

S3 Table Landscape Units defined by cluster analysis and used to randomly allocate sampling transects and sampling point counts. (DOCX)

S4 Table Species observed during the winter (W) and the breeding season (B). For each species is indicated if it is listed in the Italian Red List; EN = Endangered, VU = Vulnerable, NT = Near Threatened, LC = Least Concern, DD = Data Deficient, NA = Not Applicable), in the Annex I of the Birds Directive 2009/147/CE, and the SPEC category.
(DOCX)
