# Supplementary material for: The Importance of Rotational Crops for Biodiversity Conservation in Mediterranean Areas
Source: PLoS One. 2016 Feb 26;11(2):e0149323. doi: 10.1371/journal.pone.0149323 (PMC4769144; doi:10.1371/journal.pone.0149323)
Supplement: S1 Table — (DOCX) [file pone.0149323.s001.docx]

**THE IMPORTANCE OF ROTATIONAL CROPS FOR BIODIVERSITY CONSERVATION IN MEDITERRANEAN AREAS**

Gianpasquale Chiatante^1*^, Alberto Meriggi^1^

^1^ Department of Earth and Environmental Sciences, University of Pavia, Via Ferrata 1, 27100, Pavia, Italy

^*^ corresponding author: harrier84@libero.it, +39 333 1868129

**S1 Table.** Land use variables used for cluster analysis and as predictors in the multiple linear regression to assessing bird diversity hotspots in Southern Italy.

| **Environmental variables (% cover)** |
| --- |
| Urban areas |
| Extractive areas |
| Irrigated crops |
| Not irrigated crops |
| Horticultures |
| Vineyards |
| Orchards |
| Olive groves |
| Meadows |
| Annual crops associated with permanent crops |
| Complex cultivation patterns |
| Arable lands with natural vegetation |
| Woodlands |
| Pastures and meadows with scattered trees |
| Pastures and natural grasslands |
| Shrublands and Mediterranean maquis |
| Transitional wood/shrubs |
| Beaches, dunes, sands |
| Bare soils and rocks |
| Sparsely vegetated areas |
| Marshes, rivers, water bodies |
| Saltmarshes and salt flats |
