# Supplementary material for: The Importance of Rotational Crops for Biodiversity Conservation in Mediterranean Areas
Source: PLoS One. 2016 Feb 26;11(2):e0149323. doi: 10.1371/journal.pone.0149323 (PMC4769144; doi:10.1371/journal.pone.0149323)
Supplement: S2 Table — Density of sampling transects (1 km/km2) and sampling points (1 point/km2) are showed as well. (DOCX) [file pone.0149323.s002.docx]

**THE IMPORTANCE OF ROTATIONAL CROPS FOR BIODIVERSITY CONSERVATION IN MEDITERRANEAN AREAS**

Gianpasquale Chiatante^1*^, Alberto Meriggi^1^

^1^ Department of Earth and Environmental Sciences, University of Pavia, Via Ferrata 1, 27100, Pavia, Italy

^*^ corresponding author: harrier84@libero.it, +39 333 1868129

**S2 Table.** Landscape Units (LU), length of transects and number of point counts carried out in the research. Density of sampling transects (1 km/km^2^) and sampling points (1 point/km^2^) are showed as well.

| **LU** | **Surface (km^2^)** | **Transects** | **Point counts** |
| --- | --- | --- | --- |
| 1 | 243 | 7.8 (1 km/31 km^2^) | 11 (1 point/22 km^2^) |
| 2 | 47 | 1.0 (1 km/45 km^2^) | 1 (1 point/47 km^2^) |
| 3 | 1389 | 46.3 (1 km/30 km^2^) | 73 (1 point/19 km^2^) |
| 4 | 430 | 14.3 (1 km/30 km^2^) | 22 (1 point/20 km^2^) |
| 5 | 1042 | 34.7 (1 km/30 km^2^) | 58 (1 point/18 km^2^) |
| 6 | 273 | 8.8 (1 km/31 km^2^) | 19 (1 point/14 km^2^) |
| 7 | 295 | 9.5 (1 km/31 km^2^) | 15 (1 point/20 km^2^) |
| 8 | 590 | 20.3 (1 km/29 km^2^) | 25 (1 point/23 km^2^) |
| 9 | 68 | 2.2 (1 km/31 km^2^) | 10 (1 point/7 km^2^) |
| 10 | 1278 | 39.9 (1 km/32 km^2^) | 67 (1 point/19 km^2^) |
