# Supplementary material for: The Importance of Rotational Crops for Biodiversity Conservation in Mediterranean Areas
Source: PLoS One. 2016 Feb 26;11(2):e0149323. doi: 10.1371/journal.pone.0149323 (PMC4769144; doi:10.1371/journal.pone.0149323)
Supplement: S3 Table — (DOCX) [file pone.0149323.s003.docx]

**THE IMPORTANCE OF ROTATIONAL CROPS FOR BIODIVERSITY CONSERVATION IN MEDITERRANEAN AREAS**

Gianpasquale Chiatante^1*^, Alberto Meriggi^1^

^1^ Department of Earth and Environmental Sciences, University of Pavia, Via Ferrata 1, 27100, Pavia, Italy

^*^ corresponding author: harrier84@libero.it, +39 333 1868129

**S3 Table.** Landscape Units defined by cluster analysis and used to randomly allocate sampling transects and sampling point counts.

| **LU** | **Surface** | **Average altitude**  **(min-max)** | **Main land use (cover %)** |
| --- | --- | --- | --- |
| LU1 Orchards | 243 km^2^ | 259 m a.s.l.  (33-560) | orchards (60.2%)  not irrigated crops (11.8%)  olive groves (11.5%) |
| LU2 Salt flats | 47 km^2^ | 0 m a.s.l. | saltmarshes and salt flats (100.0%) |
| LU3 Olive groves | 1389 km^2^ | 192 m a.s.l.  (0-463) | olive groves (73.9%) |
| LU4 Not irrigated crops and natural grasslands | 430 km^2^ | 473 m a.s.l.  (14-679) | pastures and natural grasslands (50.5%)  not irrigated crops (33.2%) |
| LU5  Not irrigated crops and olive groves | 1042 km^2^ | 274 m a.s.l.  (0-632) | not irrigated crops (36.2%)  olive groves (23.9%)  urban areas (10.6%) |
| LU6 Not irrigated crops and woodland | 273 km^2^ | 421 m a.s.l.  (103-665) | woodland (50.1%)  not irrigated crops (24.6%) |
| LU7 Urban areas | 295 km^2^ | 132 m a.s.l.  (0-633) | urban areas (66.7%)  olive groves (10.9%) |
| LU8 Vineyards and olive groves | 590 km^2^ | 124 m a.s.l.  (0-558) | vineyards (47.3%)  olive groves (22.1%)  not irrigated crops (15.6%) |
| LU9 Irrigated crops | 68 km^2^ | 121 m a.s.l.  (0-253) | irrigated crops (53.5%)  vineyards (16.4%)  olive groves (10.6%) |
| LU10 Not irrigated crops | 1278 km^2^ | 400 m a.s.l.  (0-675) | not irrigated crops (78.7%) |
