# Supplementary material for: The Importance of Rotational Crops for Biodiversity Conservation in Mediterranean Areas
Source: PLoS One. 2016 Feb 26;11(2):e0149323. doi: 10.1371/journal.pone.0149323 (PMC4769144; doi:10.1371/journal.pone.0149323)
Supplement: S4 Table — For each species is indicated if it is listed in the Italian Red List; EN = Endangered, VU = Vulnerable, NT = Near Threatened, LC = Least Concern, DD = Data Deficient, NA = Not Applicable), in the Annex I of the Birds Directive 2009/147/CE, and the SPEC category. (DOCX) [file pone.0149323.s004.docx]

**THE IMPORTANCE OF ROTATIONAL CROPS FOR BIODIVERSITY CONSERVATION IN MEDITERRANEAN AREAS**

Gianpasquale Chiatante^1*^, Alberto Meriggi^1^

^1^ Department of Earth and Environmental Sciences, University of Pavia, Via Ferrata 1, 27100, Pavia, Italy

^*^ corresponding author: harrier84@libero.it, +39 333 1868129

**S4 Table.** Species observed during the winter (W) and the breeding season (B). For each species is indicated if it is listed in the Italian Red List (Peronace et al. 2012; EN = Endangered, VU = Vulnerable, NT = Near Threatened, LC = Least Concern, DD = Data Deficient, NA = Not Applicable), in the Annex I of the Birds Directive 2009/147/CE, and the SPEC category.

| **N** | **Species** | **W** | **B** | **Red List** | **Annex I** | **SPEC** |
| --- | --- | --- | --- | --- | --- | --- |
|  | Common Shelduck (*Tadorna tadorna*) | X | X | VU |  | Non-SPEC |
|  | Eurasian Wigeon (*Anaspenelope*) | X |  | NA |  | Non-SPEC |
|  | Gadwall (*Anasstrepera*) | X |  | VU |  | 3 |
|  | Eurasian Teal (*Anas crecca*) | X |  | EN |  | Non-SPEC |
|  | Mallard (*Anasplatyrhynchos*) | X | X | LC |  | Non-SPEC |
|  | Northern Pintail (*Anasacuta*) | X |  | NA |  | 3 |
|  | Northern Shoveler (*Anasclypeata*) | X |  | VU |  | 3 |
|  | Common Pochard (*Aythyaferina*) | X |  | EN |  | 2 |
|  | Red-breasted Merganser (*Mergusserrator*) | X |  |  |  | Non-SPEC |
|  | Common Quail (*Coturnixcoturnix*) |  | X | DD |  | 3 |
|  | Great Cormorant (*Phalacrocoraxcarbo*) | X |  | LC |  | Non-SPEC |
|  | Little Bittern (*Ixobrychusminutus*) |  | X | VU | X | 3 |
|  | Little Egret (*Egretta garzetta*) | X |  | LC | X | Non-SPEC |
|  | Great Egret (*Ardea alba*) | X |  | NT | X | Non-SPEC |
|  | Grey Heron (*Ardeacinerea*) | X |  | LC |  | Non-SPEC |
|  | Eurasian Spoonbill (*Platalea leucorodia*) | X |  | VU | X | 2 |
|  | Greater Flamingo (*Phoenicopterusroseus*) | X |  | LC | X | 3 |
|  | Great Crested Grebe (*Podicepscristatus*) |  | X | LC |  |  |
|  | European Honey Buzzard (*Pernisapivorus*) |  | X | LC | X |  |
|  | Black Kite (*Milvusmigrans*) | X | X | NT | X | 3 |
|  | Red Kite (*Milvusmilvus*) | X | X | VU | X | 2 |
|  | Short-toed Snake Eagle (*Circaetusgallicus*) |  | X | VU | X | 3 |
|  | Western Marsh Harrier (*Circus aeruginosus*) | X |  | VU | X | Non-SPEC |
|  | Hen Harrier (*Circus cyaneus*) | X |  | NA | X | 3 |
|  | Eurasian Sparrowhawk (*Accipiter nisus*) | X | X | LC |  | Non-SPEC |
|  | Common Buzzard (*Buteobuteo*) | X | X | LC |  | Non-SPEC |
|  | Lesser Kestrel (*Falco naumanni*) |  | X | LC | X | 1 |
|  | Common Kestrel (*Falco tinnunculus*) | X | X | LC |  | 3 |
|  | Merlin (*Falco columbarius*) | X |  |  | X | Non-SPEC |
|  | Peregrine Falcon (*Falco peregrinus*) | X | X | LC | X | Non-SPEC |
|  | Water Rail (*Rallusaquaticus*) | X |  | LC |  | Non-SPEC |
|  | Common Moorhen (*Gallinulachloropus*) | X | X | LC |  | Non-SPEC |
|  | Eurasian Coot (*Fulica atra*) | X | X | LC |  | Non-SPEC |
|  | Black-winged Stilt (*Himantopushimantopus*) |  | X | LC | X |  |
|  | Pied Avocet (*Recurvirostra avosetta*) | X | X | LC | X | Non-SPEC |
|  | Eurasian Stone-curlew (*Burhinusoedicnemus*) | X | X | VU | X | 3 |
|  | Collared Pratincole (*Glareolapratincola*) |  | X | EN | X | 3 |
|  | Common Ringed Plover (*Charadriushiaticula*) | X |  |  |  | Non-SPEC |
|  | Kentish Plover (*Charadriusalexandrinus*) | X | X | EN | X | 3 |
|  | European Golden Plover (*Pluvialisapricaria*) | X |  |  | X | Non-SPEC |
|  | Grey Plover (*Pluvialis squatarola*) | X |  |  |  | Non-SPEC |
|  | Northern Lapwing (*Vanellusvanellus*) | X |  | LC |  | 2 |
|  | Sanderling (*Calidris alba*) | X |  |  |  | Non-SPEC |
|  | Little Stint (*Calidrisminuta*) | X |  |  |  | Non-SPEC |
|  | Dunlin (*Calidris alpina*) | X |  |  |  | 3 |
|  | Ruff (*Philomachuspugnax*) | X |  |  | X | 2 |
|  | Common Snipe (*Gallinago gallinago*) | X |  | NA |  | 3 |
|  | Eurasian Woodcock (*Scolopax rusticola*) | X |  | DD |  | 3 |
|  | Black-tailed Godwit (*Limosalimosa*) | X |  | EN |  | 2 |
|  | Eurasian Curlew (*Numenius arquata*) | X |  | NA |  | 2 |
|  | Common Sandpiper (*Actitis hypoleucos*) | X |  | NT |  | 3 |
|  | Green Sandpiper (*Tringa ochropus*) | X |  |  |  | Non-SPEC |
|  | Spotted Redshank (*Tringa erythropus*) | X |  |  |  | 3 |
|  | Wood Sandpiper (*Tringa glareola*) | X |  |  | X | 3 |
|  | Common Redshank (*Tringa totanus*) | X |  | LC |  | 2 |
|  | Ruddy Turnstone (*Arenaria interpres*) | X |  |  |  | Non-SPEC |
|  | Slender-billed Gull (*Chroicocephalusgenei*) | X | X | LC | X | 3 |
|  | Black-headed Gull (*Chroicocephalusridibundus*) | X |  | LC |  | Non-SPEC |
|  | Mediterranean Gull  (*Ichthyaetus melanocephalus*) | X | X | LC | X | Non-SPEC |
|  | Audouin’s Gull (*Ichthyaetus audouinii*) | X |  | NT | X | 1 |
|  | Mew Gull (*Laruscanus*) | X |  |  |  | 2 |
|  | Lesser Black-backed Gull (*Larusfuscus*) | X |  |  |  | Non-SPEC |
|  | Yellow-legged Gull (*Larusmichahellis*) | X | X | LC |  | Non-SPEC |
|  | European Herring Gull (*Larusargentatus*) | X |  |  |  | Non-SPEC |
|  | Caspian Gull (*Larus cachinnans*) | X |  |  |  | Non-SPEC |
|  | Great Black-backed Gull (*Larusmarinus*) | X |  |  |  | Non-SPEC |
|  | Little Tern (*Sternulaalbifrons*) |  | X | EN | X | 3 |
|  | Gull-billed Tern (*Gelochelidonnilotica*) |  | X | NT | X | 3 |
|  | Sandwich Tern (*Thalasseus sandvicensis*) | X | X | VU | X | 2 |
|  | Common Wood Pigeon (*Columba palumbus*) | X | X | LC |  | Non-SPEC |
|  | Eurasian Collared Dove (*Streptopelia decaocto*) | X | X | LC |  | Non-SPEC |
|  | European Turtle Dove (*Streptopelia turtur*) |  | X | LC |  | 3 |
|  | Rose-ringed Parakeet (*Psittaculakrameri*) | X | X |  |  | Non-SPEC |
|  | Monk Parakeet (*Myiopsitta monachus*) | X | X |  |  | Non-SPEC |
|  | Great Spotted Cuckoo (*Clamatorglandarius*) |  | X | EN |  |  |
|  | Common Cuckoo (*Cuculuscanorus*) |  | X | LC |  |  |
|  | Western Barn Owl (*Tyto alba*) | X | X | LC |  | 3 |
|  | Eurasian Scops Owl (*Otusscops*) | X | X | LC |  | 2 |
|  | Little Owl (*Athene noctua*) | X | X | LC |  | 3 |
|  | Long-eared Owl (*Asiootus*) | X | X | LC |  | Non-SPEC |
|  | European Nigthjar (*Caprimulguseuropaeus*) |  | X | LC | X | 2 |
|  | Common Swift (*Apus apus*) |  | X | LC |  |  |
|  | Pallid Swift (*Apus pallidus*) |  | X | LC |  |  |
|  | Alpine Swift (*Tachymarptis melba*) |  | X | LC |  |  |
|  | Common Kingfisher (*Alcedo atthis*) | X | X | LC | X | 3 |
|  | European Bee-eater (*Meropsapiaster*) |  | X | LC |  | 3 |
|  | European Roller (*Coracias garrulus*) |  | X | VU | X | 2 |
|  | Eurasian Hoopoe (*Upupaepops*) |  | X | LC |  | 3 |
|  | Eurasian Wryneck (*Jynx torquilla*) | X | X | EN |  | 3 |
|  | European Green Woodpecker (*Picusviridis*) |  | X | LC |  | 2 |
|  | Great Spotted Woodpecker (*Dendrocopos major*) | X | X | LC |  | Non-SPEC |
|  | Calandra Lark (*Melanocorypha calandra*) | X | X | VU | X | 3 |
|  | Greater Short-toed Lark  (*Calandrellabrachydactyla*) |  | X | EN | X | 3 |
|  | Crested Lark (*Galerida cristata*) | X | X | LC |  | 3 |
|  | Woodlark (*Lullula arborea*) | X | X | LC | X | 2 |
|  | Eurasian Skylark (*Alauda arvensis*) | X | X | VU |  | 3 |
|  | Tawny Pipit (*Anthus campestris*) |  | X | LC | X | 3 |
|  | Meadow Pipit (*Anthus pratensis*) | X |  | NA |  | Non-SPEC |
|  | Water Pipit (*Anthus spinoletta*) | X |  | LC |  | Non-SPEC |
|  | Western Yellow Wagtail (*Motacillaflava*) |  | X | VU |  | Non-SPEC |
|  | Grey Wagtail (*Motacilla cinerea*) | X | X | LC |  | Non-SPEC |
|  | White Wagtail (*Motacilla alba*) | X | X | LC |  | Non-SPEC |
|  | Sand Martin (*Riparia riparia*) |  | X | VU |  | 3 |
|  | Barn Swallow (*Hirundo rustica*) |  | X | LC |  | Non-SPEC |
|  | Common House Martin (*Delichonurbicum*) |  | X | NT |  | 3 |
|  | Red-rumped Swallow (*Cecropisdaurica*) |  | X | VU |  |  |
|  | Eurasian Wren (*Troglodytes troglodytes*) | X | X | LC |  | Non-SPEC |
|  | Dunnock (*Prunella modularis*) | X |  | LC |  | Non-SPEC |
|  | European Robin (*Erithacusrubecula*) | X | X | LC |  | Non-SPEC |
|  | Common Nightingale (*Lusciniamegarhynchos*) |  | X | LC |  |  |
|  | Common Redstart (*Phoenicurusphoenicurus*) |  | X | LC |  | Non-SPEC |
|  | Black Redstart (*Phoenicurus ochruros*) | X |  | LC |  | Non-SPEC |
|  | European Stonechat (*Saxicola rubicola*) | X | X | VU |  | Non-SPEC |
|  | Black-eared Wheatear (*Oenanthehispanica*) |  | X | EN |  | 2 |
|  | Blue Rock Thrush (*Monticola solitarius*) | X | X | LC |  | 3 |
|  | Common Blackbird (*Turdus merula*) | X | X | LC |  | Non-SPEC |
|  | Fiedlfare (*Turdus pilaris*) | X |  | NT |  | Non-SPEC |
|  | Song Thrush (*Turdus philomelos*) | X |  | LC |  | Non-SPEC |
|  | Redwing (*Turdus iliacus*) | X |  | NA |  | Non-SPEC |
|  | Mistle Thrush (*Turdus viscivorus*) | X | X | LC |  | Non-SPEC |
|  | Cetti’s Warbler (*Cettia cetti*) | X | X | LC |  | Non-SPEC |
|  | Zitting Cisticola (*Cisticola juncidis*) | X | X | LC |  | Non-SPEC |
|  | Moustached Warbler  (*Acrocephalus melanopogon*) | X |  | VU | X | Non-SPEC |
|  | Eurasian Reed Warbler  (*Acrocephalusscirpaceus*) |  | X | LC |  |  |
|  | Eurasian Blackcap (*Sylvia atricapilla*) | X | X | LC |  | Non-SPEC |
|  | Common Whitethroat (*Sylvia communisi)* |  | X | LC |  |  |
|  | Spectacled Warbler (*Sylvia conspicillata*) |  | X | LC |  |  |
|  | Subalpine Warbler (*Sylvia cantillans*) |  | X | LC |  |  |
|  | Sardinian Warbler (*Sylvia melanocephala*) | X | X | LC |  | Non-SPEC |
|  | Common Chiffchaff (*Phylloscopus collybita*) | X | X | LC |  | Non-SPEC |
|  | Goldcrest (*Regulusregulus*) | X |  | NT |  | Non-SPEC |
|  | Common Firecrest (*Regulus ignicapilla*) | X |  | LC |  | Non-SPEC |
|  | Long-tailed Tit (*Aegithaloscaudatus*) | X | X | LC |  | Non-SPEC |
|  | Eurasian Blue Tit (*Cyanistescaeruleus*) | X | X | LC |  | Non-SPEC |
|  | Great Tit (*Parus major*) | X | X | LC |  | Non-SPEC |
|  | Short-toed Treecreeper (*Certhiabrachydactyla*) | X | X | LC |  | Non-SPEC |
|  | Eurasian Golden Oriole (*Oriolusoriolus*) |  | X | LC |  | Non-SPEC |
|  | Eurasian Pendulin Tit (*Remizpendulinus*) | X | X | VU |  | Non-SPEC |
|  | Great Grey Shrike (*Laniusexcubitor*) | X |  |  |  | 3 |
|  | Red-backed Shrike (*Laniuscollurio*) |  | X | VU | X | 3 |
|  | Lesser Grey Shrike (*Lanius minor*) |  | X | VU | X | 2 |
|  | Woodchat Shrike (*Lanius senator*) |  | X | EN |  | 2 |
|  | Eurasian Jay (*Garrulus glandarius*) | X | X | LC |  | Non-SPEC |
|  | Eurasian Magpie (*Pica pica*) | X | X | LC |  | Non-SPEC |
|  | Western Jackdaw (*Coloeus monedula*) | X | X | LC |  | Non-SPEC |
|  | Hooded Crow (*Corvus cornix*) | X | X | LC |  | Non-SPEC |
|  | Northern Raven (*Corvus corax*) | X | X | LC |  | Non-SPEC |
|  | Common Starling (*Sturnus vulgaris*) | X | X | LC |  | 3 |
|  | Italian Sparrow (*Passer italiae*) | X | X | VU |  | 3 |
|  | Spanish Sparrow (*Passer hispaniolensis*) | X | X | VU |  | Non-SPEC |
|  | Eurasian Tree Sparrow (*Passer montanus*) | X | X | VU |  | 3 |
|  | Rock Sparrow (*Petronia petronia*) | X | X | LC |  | Non-SPEC |
|  | Common Chaffinch (*Fringilla coelebs*) | X | X | LC |  | Non-SPEC |
|  | Brambling (*Fringilla montifringilla*) | X |  | NA |  | Non-SPEC |
|  | European Serin (*Serinus serinus*) | X | X | LC |  | Non-SPEC |
|  | European Greenfinch (*Chloris chloris*) | X | X | NT |  | Non-SPEC |
|  | European Goldfinch (*Carduelis carduelis*) | X | X | NT |  | Non-SPEC |
|  | Eurasian Siskin (*Spinus spinus*) | X |  | LC |  | Non-SPEC |
|  | Common Linnet (*Linaria cannabina*) | X | X | NT |  | 2 |
|  | Hawfinch (*Coccothraustescoccothraustes*) | X |  | LC |  | Non-SPEC |
|  | Cirl Bunting (*Emberiza cirlus*) | X | X | LC |  | Non-SPEC |
|  | Black-headed Bunting  (*Emberiza melanocephala*) |  | X | NT |  | 2 |
|  | Common Reed Bunting (*Emberiza schoeniclus*) | X |  | NT |  | Non-SPEC |
|  | Corn Bunting (*Emberiza calandra*) | X | X | LC |  | 2 |
